# Duplication and diversification of the *LEAFY HULL STERILE1 and Oryza sativa MADS5 SEPALLATA *lineages in graminoid Poales

**DOI:** 10.1186/2041-9139-3-4

**Published:** 2012-02-17

**Authors:** Ashley R Christensen, Simon T Malcomber

**Affiliations:** 1 Department of Biological Sciences, California State University - Long Beach, 1250 Bellflower Boulevard, Long Beach, CA 90840, USA; 2 Agensys, Inc., an Affiliate of Astellas Pharma Inc., 1545 17th Street, Santa Monica, CA 90404, USA

**Keywords:** grasses, *LHS1*, MADS-box, *OsMADS5*, Poaceae, neofunctionalization, subfunctionalization

## Abstract

**Background:**

Gene duplication and the subsequent divergence in function of the resulting paralogs via subfunctionalization and/or neofunctionalization is hypothesized to have played a major role in the evolution of plant form. The *LEAFY HULL STERILE1 (LHS1) SEPALLATA *(*SEP*) genes have been linked with the origin and diversification of the grass spikelet, but it is uncertain 1) when the duplication event that produced the *LHS1 *clade and its paralogous lineage *Oryza sativa MADS5 (OSM5) *occurred, and 2) how changes in gene structure and/or expression might have contributed to subfunctionalization and/or neofunctionalization in the two lineages.

**Methods:**

Phylogenetic relationships among 84 *SEP *genes were estimated using Bayesian methods. RNA expression patterns were inferred using *in situ *hybridization. The patterns of protein sequence and RNA expression evolution were reconstructed using maximum parsimony (MP) and maximum likelihood (ML) methods, respectively.

**Results:**

Phylogenetic analyses mapped the *LHS1/OSM5 *duplication event to the base of the grass family. MP character reconstructions estimated a change from cytosine to thymine in the first codon position of the first amino acid after the *Zea mays MADS3 *(*ZMM3*) domain converted a glutamine to a stop codon in the *OSM5 *ancestor following the *LHS1/OSM5 *duplication event. RNA expression analyses of *OSM5 *co-orthologs in *Avena sativa, Chasmanthium latifolium, Hordeum vulgare, Pennisetum glaucum*, and *Sorghum bicolor *followed by ML reconstructions of these data and previously published analyses estimated a complex pattern of gain and loss of *LHS1 *and *OSM5 *expression in different floral organs and different flowers within the spikelet or inflorescence.

**Conclusions:**

Previous authors have reported that rice OSM5 and LHS1 proteins have different interaction partners indicating that the truncation of OSM5 following the *LHS1/OSM5 *duplication event has resulted in both partitioned and potentially novel gene functions. The complex pattern of *OSM5 *and *LHS1 *expression evolution is not consistent with a simple subfunctionalization model following the gene duplication event, but there is evidence of recent partitioning of *OSM5 *and *LHS1 *expression within different floral organs of *A. sativa, C. latifolium, P. glaucum *and *S. bicolor*, and between the upper and lower florets of the two-flowered maize spikelet.

## Background

The diversification of paralogs following developmental gene duplication events is hypothesized to have played a major role in the evolution of morphological form [1-3]. Three general fates are hypothesized for duplicated gene products [2]. In the bulk of cases one of the gene products is predicted to accumulate mutations, become a pseudogene and be purged from the genome (pseudogenization). Alternatively, in a limited number of cases it is hypothesized that either changes to regulatory regions will result in the ancestral gene function being partially or completely partitioned between the two duplicates (subfunctionalization), or changes to regulatory domains and/or coding regions will result in a new gene function in one or both of the duplicates (neofunctionalization). Either the neofunctionalized developmental gene copies alone, or a combination of sub- and neofunctionalized copies are hypothesized to provide the raw genetic material necessary for the production of novel morphological structures.

The monocot clade comprises approximately 56,000 flowering plants, including such morphologically divergent lineages as orchids, palms, gingers, and grasses. Most monocot inflorescences contain one-many flowers, comprised of two whorls of three tepals, six stamens, and a three-carpellate pistil [4]. In contrast, grass inflorescences have, depending on the species, 1-40 flowers (or florets) collected into novel structures called grass spikelets [5]. At the base of each grass spikelet are two bract-like structures called glumes and each grass floret typically contains a lemma, a palea, two lodicules, three stamens, and a pistil. Sister to the grasses is a lineage comprised of the small families Joinvilleaceae and Ecdeiocoleaceae [6]. Joinvilleaceae and Ecdeiocoleaceae, in addition to other non-grass members of the graminoid Poales clade, have typical monocot flowers suggesting that a series of profound developmental and genetic changes occurred early in the history of the Poaceae lineage to produce grass spikelets, glumes, lemmas, paleas and lodicules typical of most taxa within the family.

The *LOFSEP SEPALLATA (SEP) *genes - containing the rice *Oryza sativa MADS1*/*LEAFY HULL STERILE1 (OsMADS1/OsLHS1), Oryza sativa MADS5 (OSM5) *and *Oryza sativa MADS34/PANICLE PHYTOMER2 *(*OsMADS34*/*OsPAP2*) genes, the petunia *FLORAL BINDING PROTEIN9 *(*PhFBP9*) and *PhFBP23 *genes, and *Arabidopsis SEP1, SEP2 *and *SEP4 *genes [7,8] - are hypothesized to have played a role in the origin and diversification of the grass spikelet [8,9]. *SEP *genes act as co-factors with ABC identity genes to help specify the identity of the different floral whorls [10,11]. Although the four *Arabidopsis SEP *(*AtSEP1-4*) genes are functionally redundant [12], at least two grass members of the *LOFSEP *clade, the rice *OsLHS1/MADS1 *and *OsPAP2/MADS34 *genes, have non-redundant roles during various stages of inflorescence development. Mutations to *OsLHS1/MADS1 *and *OsPAP2/MADS34 *are the basis for three named mutants: the *OsLHS1/MADS1 *mutants *leafy hull sterile1 *and *naked seed rice *(*nsr*), and the *OsPAP2/MADS34 *mutant *panicle phytomer2 *(*Ospap2*). Both *Oslhs1 *and *Osnsr *are characterized by having leafy lemmas and paleas, leafy lodicules that resemble the lemma and palea, a decreased number of stamens, and occasionally, an extra pistil or floret [13,14]. *Ospap2-1 *mutants have more inflorescence branches, a disorganized arrangement of branches and spikelets, and elongated glumes and sterile lemmas compared to WT, but no obvious abnormalities in the lemma, palea, lodicules, stamens and pistil [15]. In a separate study, rice *osmads34 *mutants were reported as displaying an increase in the number of primary inflorescence branches, fewer secondary branches, fewer spikelets, and elongated sterile lemmas [16]. The difference in the number of secondary branches between the two studies is likely due to the different genetic backgrounds and type of mutations [16], but both studies agree on roles for the gene in regulating inflorescence branch number and sterile lemma morphology. Mutants of the remaining rice *LOFSEP *gene, *osmads5*, have only mild phenotypes compared to the WT plants, with lodicules attached to the palea and lemma [17]. Silencing of the rice *LHS1, OsMADS5 *and the two *SEP3 *co-orthologs, *OsMADS7 *and *OsMADS8 *is sufficient to convert floral organs into leaf-like structures suggesting the four genes in concert perform the vast majority of *SEP *or E-class function during floral development [18].

Phylogenetic analyses of the monocot *LOFSEP *clade reconstruct the *LHS1 *and *OSM5 *clades as sister taxa, and the *PAP2 *clade as sister to a combined *LHS1/OSM5 *clade [3,19]. These analyses suggest that both the *LHS1/OSM5 *and the *LHS1+OSM5/PAP2 *gene duplications occurred near the base of the grass family [3,19], but additional sequences from early diverging grasses and other graminoid Poales are necessary to both test this hypothesis and provide a framework to investigate evidence of gene sub- and/or neofunctionalization within the lineage.

Expression patterns provide evidence of potential subfunctionalization and neofunctionalization between different paralogs [2]. Of the grass *LOFSEP *genes, expression patterns within the *LHS1 *clade are the most extensively studied and have been detected in the floral and spikelet meristems, in lemmas and paleas, and variously in the other floral organs, but not in glumes (except in wheat) [9,20,21]. Reinheimer *et al*. [20] used maximum parsimony (MP) character reconstructions and estimated an ancestral *LHS1 *expression pattern in the lemma, palea and carpels. *LHS1 *expression in lodicules was reconstructed to have been gained independently in the barley (*Hordeum vulgare*) lineage and at the base of the centothecoid and panicoid clade. *LHS1 *expression in stamens was gained independently in the maize (*Zea mays*) and *Chasmanthium latifolium *lineages, and *LHS1 *expression in the gynoecium was inferred to have been lost independently in the *Eleusine indica *and Paniceae (*Pennisetum glaucum *and *Megathyrsus maximus*) lineages. Based on this comparative analysis *LHS1 *was also hypothesized to act as a selector gene [22], specifying the terminal floret, in species whose spikelets mature basipetally, but not in species whose spikelets mature acropetally [9,20]. This complex pattern of *LHS1 *expression pattern in grasses led Malcomber and Kellogg [9] to hypothesize that differences in *LHS1 *expression pattern coupled with changes in interacting partner could have played a major role in the origin and diversification of the grass spikelet. Expression profiles of *PAP2 *and *OSM5 *co-orthologs in grasses are currently limited, but rice *OsMADS34/PAP2 *is broadly expressed in the floret meristem early in development, before becoming localized to the lemma, palea and stamens later in development [23]. Rice *OSM5 *is expressed in stamens and carpels, but not lemmas and paleas [24] and expression of the maize *OSM5 *co-ortholog, *ZmM3*, is restricted to the lower floret [25].

Adding additional expression data from the *OSM5 *lineage and investigating the evolution of *LOFSEP *expression patterns within a phylogenetic context will not only provide new insights into possible evidence of sub- or neo-functionalization in the clades, but also provide insights into the patterns of gene evolution following duplication events.

## Methods

### Plant Materials

All plants were grown in greenhouses under natural light at 20 to 28**°**C with twice daily watering and regular fertilizing at California State University - Long Beach.

### Gene Isolation

Total RNA was extracted from inflorescences, leaves and vegetative apices and cDNA synthesized as described by Woods *et al*. [26]. *LOFSEP *genes were isolated using either a semi-nested RACE PCR approach anchored using a polyT + adaptor primer (5'-CCG GAT CCT CTA GAG CGG CCG CTT TTT TTT TTT TTT TTT V-3') or using species specific primers with the reverse primer located in the 3' UTR (Additional File 1). All primers were designed using Primaclade [27]. PCR products were cleaned using silica spin column (Epoch Biolabs, Sugar Land, TX, US) and sub-cloned using a pGEM-T vector kit (Promega, Madison, WI, US). Both DNA strands were then sequenced using standard dideoxy sequencing protocols. The 35 new *LOFSEP *sequences isolated during this research have been deposited with the GenBank Data Libraries [Genbank: JN661596-JN661630]

### Expression Analysis

RNA *in situ *hybridization of *OSM5 *co-orthologs in *Avena sativa, C. latifolium, H. vulgare, P. glaucum *and *Sorghum bicolor *were conducted on developing spikelets using 3' UTR probes derived from Reverse Transcription Polymerase Chain Reaction (RT-PCR) gene fragments as described by Malcomber and Kellogg [9]. Slides were imaged using an Olympus BX51 compound microscope with an Olympus DX4 digital camera. Hybridizations were repeated at least three times using different dissected and embedded materials to check for consistency. Images were cropped and adjusted for white balance using Adobe Photoshop CS5. Sense negative control hybridizations all showed no staining (data not shown).

### Phylogenetic and molecular evolutionary analyses

*LOFSEP *genes were identified by Basic Local Alignment *Search *Tool (BLAST) searches at the National Center for Biotechnology Information (NCBI) (http://www.ncbi.nlm.nih.gov) and PlantGDB (http://www.plantgdb.org) using full-length coding regions of the rice *OsLHS1/MADS1 (Os03g11614), OsMADS5 (Os06g06750) *and *OsPAP2/MADS34 *(*Os03g54170*) sequences as search seeds. Full-length sequences were assembled and translated into conceptual amino acids using Mesquite 2.74 [28] and then aligned using multiple sequence comparison by log-expectation (MUSCLE) [29] before being manually adjusted using MacClade 4.0 [30]. As in previous analyses of the *SEP *gene family [3,7,9,20], no regions were considered unalignable and excluded from subsequent analysis (See Additional File 2 for the phylogenetic data set). Bayesian phylogenetic analyses of the full-length *LOFSEP *data set using MrBayes 3.1.2 [31] were run on the Grethor parallel processing cluster at the University of Missouri - St. Louis and consisted of two separate searches of ten million generations using flat priors and the General Time Reversible (GTR) model of sequence evolution with invariant sites and gamma distributed rates partitioned according to codon position (GTR + I + SS). Trees were sampled every 1,000 generations and convergence between the two runs was determined by examining the average standard deviation of the split frequencies. After ten million generations the split frequencies between the two runs was 0.006531. After convergence had been assured the first 25% of trees were removed as burn-in and clade credibility values estimated using MrBayes.

The Shimodaira-Hasegawa test [32] for significance between the best maximum likelihood (ML) tree identified in the Bayesian search and the best tree consistent with the *LHS1/OSM5 *duplication mapping at the base of the combined Bambusoideae, Ehrhartoideae and Pooideae (BEP) and Panicoideae, Aristidoideae, Chloridoideae, Centothecoideae, Micrairoideae, Arundinoideae and Danthonioideae (PACCMAD) clade and after the divergence of Pharioideae was conducted in PAUP* 4.0 [33].

MP and ML character reconstruction analyses were conducted using Mesquite version 2.74 [28]. To facilitate comparisons among the different taxa we followed Whipple *et al*. [34], Sajo and Rudall [35] and Preston *et al*. [36] and considered: 1) bract 6 of the early diverging grass *Streptochaeta angustifolia *as homologous to one of the outer tepals in other monocots, a sepal in eudicots and the lemma in spikelet grasses, 2) bracts 7 and 8 of *S. angustifolia *as homologous to the remaining two outer tepals in other monocots, sepals in eudicots, and the palea of spikelet grasses, and 3) bracts 9-11 of *S. angustifolia *as homologous to the inner tepals in other monocots, petals in eudicots, and lodicules in spikelet grasses.

Tests for sites potentially under positive selection within the *LOFSEP *alignment utilized the best ML topology from the Bayesian search and the CODEML program within the Phylogenetic Analysis Using Maximum Likelihood (PAML) package [37] on the Grethor parallel processing cluster at University of Missouri - St Louis. Evidence of positive selection at particular codons was tested using the nested codon models M0 and M3, M1a and M2a, and M7 and M8 [38-40] with significance determined using a standard likelihood ratio test (LRT) statistic against a χ2 distribution with two degrees of freedom.

**Figure 1:**
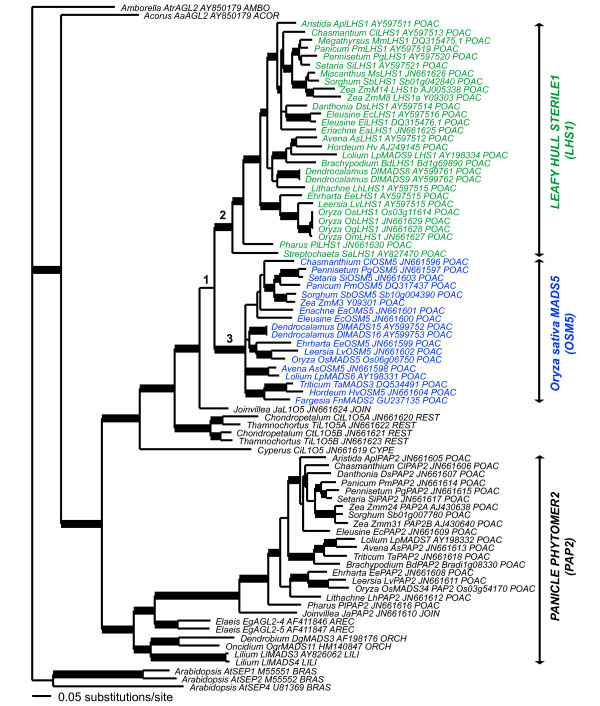
**Bayesian consensus phylogram of 84 *LOFSEP *genes**. Relationships estimated using the General Time Reversible (GTR) model, invariant sites and site specific rates partitioned according to codon position (GTR + I + SS). Phylogram rooted using *Amborella AtAGL2 AY850179*. Bold branches are supported by posterior probability ≥0.95. Family abbreviations: ACOR = Acoraceae, AMBO = Amborellaceae, AREC = Arecaceae, BRAS = Brassicaceae, CYPE = Cyperaceae, JOIN = Joinvilleaceae, LILI = Liliaceae, ORCH = Orchidaceae, POAC = Poaceae, REST = Restionaceae. Species abbreviations: *Aa = Acorus americanus, Al = Aristida longiseta, As = Avena sativa, At = Arabidopsis thaliana, Atr = Amborella trichopoda, Bd = Brachypodium distachyon, Ci = Cyperus involucratus, Cl = Chasmanthium latifolium, Ct = Chondropetalum tectorum, Dl = Dendrocalamus latiflorus, Dg = Dendrobium grex Madame Thong-In, Ds = Danthonia spicata, Ea = Eriachne aristata, Ec = Eleusine coracana, Ee = Ehrharta erecta, Eg = Elaeis guianensis, Fn = Fargesia nitida, Hv = Hordeum vulgare, Ja = Joinvillea ascendens, Lh = Lithachne humilis, Ll = Lilium longiflorum, Lp = Lolium perenne, Lv = Leersia virginica, Mm = Megathyrsus maximus, Ms = Miscanthus sinensis, Ob = Oryza barthii, Og = Oryza glaberrima, Om = Oryza meridionalis, Os = Oryza sativa, Pg = Pennisetum glaucum, Pl = Pharus latifolius, Pm = Panicum maximum, Sa = Streptochaeta angustifolia, Sb = Sorghum bicolor, Si = Setaria italica, Ta = Triticum aestivum, Ti = Thamnochortus insignis, Zm = Zea mays*. Green = *LEAFY HULL STERILE1 *(*LHS1*) clade, Blue branches = *Oryza sativa MADS5 *(*OSM5*) clade. Branch 1 = *LHS1+OSM5 *clade, Branch 2 = *LHS1 *clade, and Branch 3 = *OSM5 *clade. Branches 1 to 3 examined in PAML analyses were tested for evidence of potential positive selection (see also Additional File 3).

We also tested for sites potentially under positive selection on the branch subtending the *LHS1 *clade, the branch subtending the *OSM5 *clade, and the branch subtending the *LHS1+OSM5 *clades (Branches 1 to 3, Figure 1) using the modified branch-site models A and B [39,40]. Model A was compared with model M1a (NearlyNeutral) and model B was compared with M3 (discrete) with two site classes in a LRT against a χ2 distribution with two degrees of freedom [39,40]

## Results

### *LOFSEP *gene duplication and loss in graminoid Poales

Our Bayesian phylogenetic analysis of 84 *LOFSEP *genes estimated well-supported grass *LHS1 *(1.00 posterior probability [PP]), *OSM5 *(1.00 PP), and *PAP2 *(1.00 PP) clades (Figure 1 and Additional File 3). Although the sister relationship of the *LHS1 *and *OSM5 *subclades was only weakly supported (0.88 PP), the sister relationship of *Joinvillea LHS1/OSM5-like *(*JaL1O5*) to the *LHS1/OsMADS5 *clade was well supported (1.00 PP). The first sequence to diverge within the *LHS1 *clade was isolated from a member of the earliest diverging lineage of grasses, *S. angustifolia *(subfamily Anomochlooideae), followed by a member of the earliest diverging grass lineage with true spikelets, *Pharus latifolia *(subfamily Pharioideae). To further investigate support for the estimated relationships we employed a Shimodaira-Hasegawa (SH) test [32] to compare the best ML tree from the Bayesian search with the best ML tree satisfying the constraint that the *LHS1/OSM5 *duplication occurred at the base of the BEP+PACCMAD clades (after the divergence of *P. latifolia*). This analysis estimated the constraint tree (-ln 29886.59) as significantly less likely than the unconstrained analysis (-ln 23530.75, *P *< 0.001). Taken together, these data suggest that the *LHS1/OSM5 *(*L1O5*) duplication event occurred at the base of the grass family.

A clade of *L1O5-like *genes from two members of the graminoid Poales family Restionaceae was sister to *JaL1O5*, grass *LHS1 *and *OSM5 *clades, consistent with organismal relationships [6]. Both sampled members of the Restionaceae, *Chondropetalum tectorum *and *Thamnochortus insignis*,, had two *L1O5 *gene copies. *C. tectorum L1O5a *(*CtL1O5a*) and *T. insignis L1O5a *(*TiL1O5a*) formed a well-supported sister relationship (1.00 PP) that was sister to a *CtL1O5b *and *TiL1O5b *clade (1.00 PP), suggesting a gene duplication at the base of the African Restionaceae clade, or perhaps deeper within the lineage. A sequence isolated from a member of the Poales family Cyperaceae, *Cyperus involucrata L1O5 *(*CiL1O5*), was sister to the Restionaceae *L1O5, JaL1O5*, and grass *LHS1*/*OsMADS5 *clade (1.00 PP) suggesting an origin for the *L1O5 LOFSEP *clade at least within Poales, and potentially deeper within monocots.

Sister to the grass *PAP2 *clade was *Joinvillea JaPAP2 *(1.00 PP). This graminoid Poales clade was, in turn, sister to a clade of two oil palm sequences (*Elias EgAGL2-4 *and *EgAGL2-5*) that together comprised a well-supported commelinoid *PAP2 *clade (1.00 PP). Sister to the commelinoid *PAP2 *clade was a well-supported clade (0.99 PP) comprised of two orchid (*Dendrobium DOMADS3 *and *Oncidium OgrMADS11*) and two lily sequences (*Lilium LlMADS3 and LlMADS4*). The Poales *L1O5 *and monocot *OsMADS34/PAP2 *clades are, in turn, sister to an *Acorus LOFSEP *gene (*AaAGL2*, 1.00 PP). *Acorus *is considered to be within the earliest diverging monocot lineage [4], suggesting that the gene duplication event to produce the *PAP2 *and *L1O5 *lineages occurred after the divergence of the Acorales, but prior to the divergence of the Liliales, Asparagales and Commelinoid clade.

### Molecular evolution of *LOFSEP *genes

Changes in an amino acid sequence could have a deleterious, neutral or potentially beneficial effect on protein function by altering binding domains, changing protein stability, or modifying protein-folding ability. We used a combination of ML molecular evolutionary and MP character reconstruction approaches to investigate the changes in amino acid sequence prior to and following the *LHS1/OSM5 *duplication event at the base of the grass family. We first used the CODEML program within the PAML package [37] to test for evidence of positive selection using the nested models M0 and M3, M1a and M2a, M7 and M8 over the length of the aligned proteins followed by the branch-site models A and B on the branches subtending the *LHS1, OSM5 *and *LHS1+OSM5 *clades (Branches 1 to 3, Figure 1). All of these analyses recovered evidence of strong purifying selection (Additional File 4).

**Figure 2:**
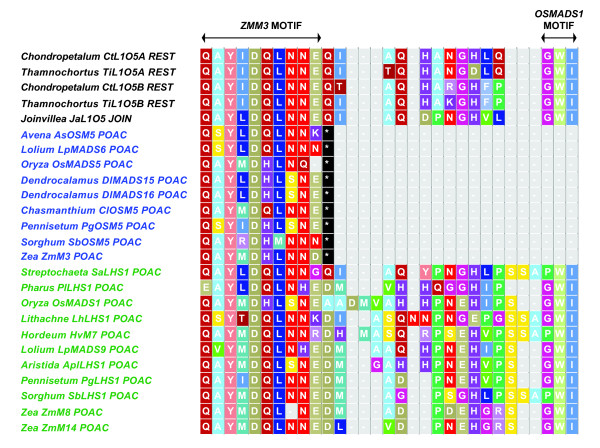
**Alignment of amino acids within the C-terminus of graminoid Poales LOFSEP proteins**. Proteins aligned using MUSCLE [29] and then manually adjusted. *OSMADS1 *and *ZMM3 *motifs identified by Vandenbussche *et al*.[41]. Green = *LEAFY HULL STERILE1 *(*LHS1*) clade, Blue branches = *Oryza sativa MADS5 *(*OSM5*) clade.

Of the four characteristic domains of MIKC-type MADS box genes, the C-terminal domain is both highly variable and critical for providing functional specificity via transcriptional activation [41]. Despite the lack of conservation of the C-terminal domain across the entire MADS-box gene family, individual gene clades often have conserved motifs [41]. Vandenbussche and colleagues [41] described two novel C-terminal motifs found in the monocot *AGL2-like *subfamily, *ZMM3 *and *OSMADS1*. We examined the sequence alignment of isolated *LOFSEP-like *proteins from members of the Restionaceae, Joinvilleaceae, and Poaceae to identify the presence or absence of the *ZMM3 *and *OSMADS1 *motifs. All sampled L1O5, LHS1, and OSM5 proteins in our analysis contained the *ZMM3 *motif. Both the L1O5 and LHS1 protein sequences also contained the *OSMADS1 *motif, but this motif was lacking among OSM5 orthologs (Figure 2).

**Figure 3:**
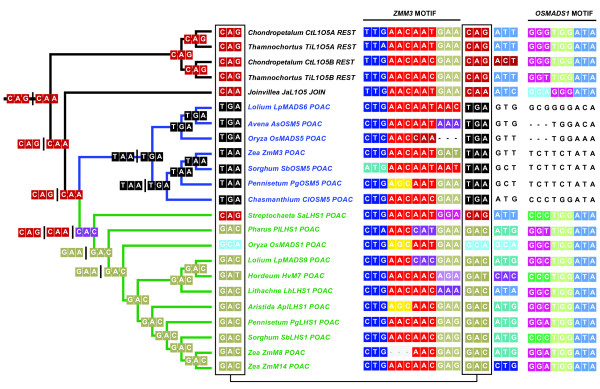
**Maximum parsimony character state reconstructions of nucleotide changes within the C-terminus of graminoid Poales LOFSEP proteins**. Reconstructed evolution of the codon immediately after the *ZMM3 *domain estimated a change from glutamine to stop codon at the base of the OSM5 lineage (left hand side). Multiple codons per branch separated by vertical lines represent equally parsimonious reconstructed amino acids. Aligned *ZMM3 *and *OSMADS1 *motifs among *LOFSEP *genes (right hand side). Colored boxes = translated amino acids, Uncolored boxes = non-coding nucleotides. Beige box = Aspartic acid, Black box = Stop, Pale Blue box = Alanine (A), Purple box = Histidine (H), Red box = Glutamine (Q). Green = *LEAFY HULL STERILE1 *(*LHS1*) clade, Blue branches = *Oryza sativa MADS5 *(*OSM5*) clade.

We then used MP character reconstruction methods to investigate nucleotide changes following the *LHS1/OSM5 *duplication event that resulted in the truncation of OSM5 protein sequences. These analyses estimated a cytosine (C) to thymine (T) nucleotide substitution in the equivalent of the first codon position of the glutamine (Q) in the *L1O5 *common ancestor at the base of the *OSM5 *clade (Figure 3). This nucleotide substitution converted the ancestral glutamine into a stop codon in the first codon after the *ZMM3 *motif resulting in the truncation of OSM5 proteins (Figure 3). The MP nucleotide reconstruction analysis estimated ambiguity in the second codon position in the first codon of the *ZMM3 *domain with an adenine (A) or guanine (G) considered equally parsimonious. Neither of these changes, however, affected the reconstructed amino acid change from glutamine to a stop codon (Figure 3).

### Heterogeneous *OSM5 *expression profiles

Comparative mRNA expression studies in MADS box gene families are powerful tools to identify potential genetic interactors and infer whether expression patterns have diverged. To investigate the pattern of expression evolution within the *OSM5 *and *LHS1 *lineages we supplemented published *LOFSEP *gene expression profiles with mRNA expression data on *OSM5 *orthologs from a sample of taxa spanning the major diversification of the family.

**Figure 4:**
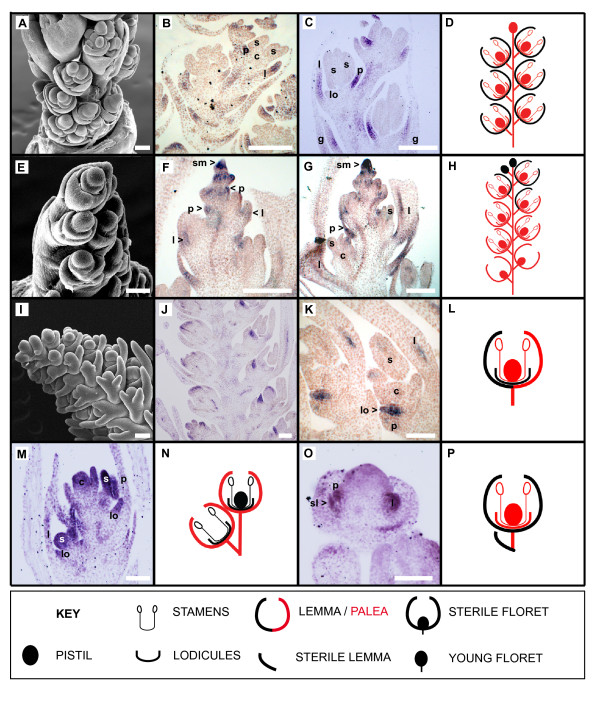
**Scanning Electron Microscopy (SEM) analysis and mRNA expression profiles of *OSM5 *co-orthologs in *Avena sativa, Chasmanthium latifolium, Hordeum vulgare, Pennisetum glaucum*, and *Sorghum bicolor *inflorescences**. Scanning electron microscopy (SEM) images of inflorescence development in *Avena sativa *(**A**), *Chasmanthium latifolium *(**E**), and *Hordeum vulgare *(**I**), mRNA *in situ *hybridization expression analyses of *OSM5 *co-orthologs in developing inflorescences of *A. sativa *(**B-C**), *C. latifolium *(**F-G**), *H. vulgare *(**J-K**), *P. glaucum *(**M**) and *S. bicolor *(**O**) using antisense digoxygenin-labeled RNA probes, and cartoons summarizing *OSM5 *expression in the different taxa (**D, H, L, N **and **P**). Developing *A. sativa *spikelets with *AsOSM5 *expression in lemmas and paleas (B-D). Developing *C. latifolium *spikelets with *ClOSM5 *expression in the spikelet meristem, lemma and palea of upper florets and no expression in the lower florets (F-H). Developing *H. vulgare *spikelets with *HvOSM5 *expression in lemmas and lodicules (J-L). *P. glaucum *spikelets with *PgOSM5 *expression in carpels, stamens and lodicules of the upper floret and stamens and lodicules of the lower spikelet (M-N). Young *S. bicolor *spikelet with *SbOSM5 *expression in the palea and lemma of the upper floret and sterile lemma of the lower floret (O-P). Abbreviations: c = carpels, l = lemma, lo = lodicule, p = palea, s = stamen, sm = spikelet meristem. Bars, 100 μm.

*A. sativa *(subfamily Pooideae) has a two- to six-flowered, acropetally maturing spikelet (Figure 4A). *A. sativa OSM5 *(*AsOSM5*) was detected in lemmas and paleas within all florets of the developing spikelet (Figure 4B-D).

*C. latifolium *(subfamily Centothecoideae) has acropetally maturing spikelets comprised of 4 to 24 florets (Figure 4E). *C. latifolium OSM5 *(*ClOSM5*) was detected in the spikelet meristem and the developing palea, and in both the lemma and palea slightly later in development (Figure 4F-H).

*H. vulgare *(subfamily Pooideae) has an indeterminate inflorescence with one-flowered spikelets clustered together in triads on short secondary inflorescence branches (Figure 4I). Only the central spikelet is bisexual, whereas the two lateral spikelets are sterile and reduced to awns. *H. vulgare OSM5 *(*HvOSM5*) was detected within the lemma and lodicules in developing florets (Figure 4J-L).

*P. glaucum *(subfamily Panicoideae) has a two-flowered spikelet that matures basipetally. The upper floret is bisexual, whereas the lower floret is staminate or sterile. *P. glaucum OSM5 *(*PgOSM5*) was detected in the lodicules, stamens and carpels of the upper floret and the stamens and lodicules of the lower floret later in development (Figure 4M-N).

*S. bicolor *(subfamily Panicoideae) has an inflorescence comprised of sessile and pedicellate basipetally maturing two-flowered spikelets. The upper floret of the sessile spikelet is bisexual, whereas the upper floret of the pedicellate spikelet is staminate or sterile. The lower floret in both the sessile and pedicellate spikelets is reduced to a sterile lemma. *S. bicolor OSM5 *(*SbOSM5*) was detected in the lemma and palea of the upper floret and the sterile lemma of the lower floret (Figure 4O-P).

### Complex patterns of *LHS1 *and *OSM5 *mRNA expression evolution

To investigate evidence of sub- or neofunctionalization following the *L1O5 *duplication event we combined our new expression data on *OSM5 *genes with published data on other *LOFSEP *genes [9,13,20-25,36,42-48] and reconstructed the pattern of evolution using ML within Mesquite.

**Figure 5:**
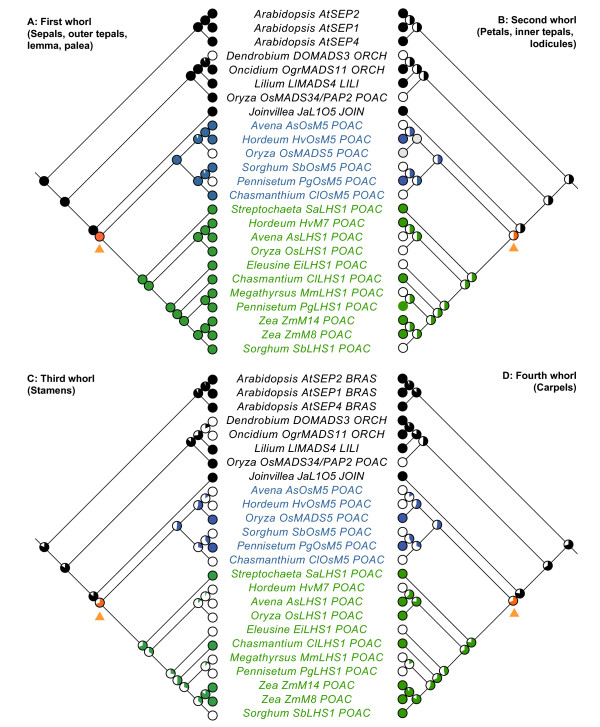
**Maximum likelihood character reconstructions of LOFSEP gene expression in flowers**. **(A) **Expression evolution in first whorl structures (sepals, outer tepals, lemma and palea). (**B**) Expression evolution in second whorl structures (petals, inner tepals and lodicules). (**C) **Expression evolution in third whorl structures (stamens). (**D**) Expression evolution in fourth whorl structures (carpels). Solid circle = presence of expression, empty circle = absence of expression, partially filled circles = reconstructed likelihood of the presence/absence of expression. Blue branches = *Oryza sativa MADS5 *(*OSM5*) clade, Green = *LEAFY HULL STERILE1 *(*LHS1*) clade, Grey = missing data, Orange = *LHS1+OSM5 *ancestor.

**Figure 6:**
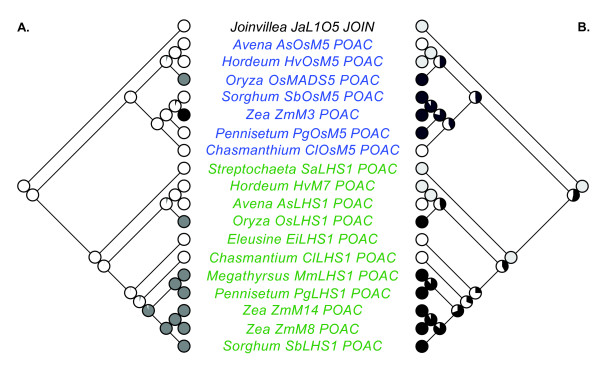
**Maximum likelihood character reconstructions of graminoid poales LOFSEP gene expression in inflorescences and spikelets**. **(A) **Expression evolution in different regions of the inflorescence and spikelet. Black = Expression restricted to the lower floret within the spikelet, Grey = Expression restricted to the upper floret in the spikelet, White = Expression present in all flowers of the inflorescence. (B) Evolution of floral maturation. Black = basipetally maturing spikelets, Grey = Flowers borne singly or spikelets comprised of a single floret, White = acropetally maturing spikelets. Green = *LEAFY HULL STERILE1 *(*LHS1*) clade, Blue branches = *Oryza sativa MADS5 *(*OSM5*) clade.

All analyses recovered complex patterns of expression evolution in the different floral structures (Figure 5) and in different flowers/florets within the inflorescence (Figure 6). *Joinvillea L1O5 *is expressed within all floral whorls and all flowers within the inflorescence [36]. *LHS1 *was expressed in the lemma and palea of all sampled grasses, whereas *OSM5 *co-orthologs were also detected in the lemma and palea of *A. sativa, C. latifolium*, and *S. bicolor*, and the lemma of *H. vulgare *(Figure 5A). The presence of *OSM5 *expression within the lemma and palea was reconstructed as likely at the base of the clade, with the loss of expression estimated to have occurred independently in the rice and *P. glaucum *lineages.

Expression within whorl 2 is more homoplasious than whorl 1 (Figure 5B). *LHS1 *was detected in second whorl structures of *S. angustifolia, H. vulgare, C. latifolium, P. glaucum *and *Z. mays *whereas *OSM5 *was only expressed in lodicules of *H. vulgare *and *P. glaucum *(whether *O. sativa OSM5 *is expressed in lodicules is unknown). The reconstructed ancestral state at the base of the *LHS1, OSM5 *and *LHS1+OSM5 *clade is ambiguous in our analyses due to homoplasy within the clade, so it is uncertain whether expression was gained in those taxa expressing *LHS1 *or *OSM5 *in whorl 2 structures, or whether expression was lost in the other taxa.

The presence of expression in stamens was reconstructed as the more likely ancestral state at the base of the *LHS1, OSM5 *and *LHS1+OSM5 *clades (Figure 5C). *LHS1 *is expressed in the stamens of *S. angustifolia, C. latifolium *and *Z. mays*, and *OSM5 *is detected in the stamens of *O. sativa *and *P. glaucum*. ML character reconstructions estimate the independent loss of *OSM5 *expression in the pooid clade (*A. sativa *and *H. vulgare*) and at the base of PACCMAD grasses followed by the subsequent gain of expression in *P. glaucum. LHS1 *expression was lost on the branch subtending the BEP+PACCMAD clade and subsequently regained independently within the *C. latifolium *and *Z. mays *lineages.

The presence of expression within carpels was reconstructed as the ancestral state at the base of the *LHS1 *and *LHS1+OSM5 *clade, and marginally more likely at the base of the *OSM5 *clade (Figure 5D). *OSM5 *expression in carpels was subsequently lost in pooid grasses (*A. sativa *and *H. vulgare*) and at the base of panicoid+centothecoid clade, but subsequently regained within the *P. glaucum *lineage. Within the *LHS1 *clade, expression in carpels was independently lost in *H. vulgare, E. indica*, and in the Paniceae (*M. maximum and P. glaucum*).

Cacharrón and colleagues [22] hypothesized that the maize *LHS1 *and *OSM5 *co-orthologs. *ZmM8, ZmM14 *and *ZmM3*, respectively, function as selector genes during inflorescence development. Expression of the maize *LHS1 *co-orthologs *ZmM8 *and *ZmM14 *were hypothesized to specify the terminal floret of the two-flowered spikelet [22], whereas expression of the *OSM5 *ortholog *ZmM3 *was hypothesized to specify the lower floret [43]. Using the expanded *L1O5, LHS1 *and *OSM5 *dataset we find no support for a subfunctionalization of *LHS1 *and *OsMADS5 *function into different flowers of the spikelet at the base of the two clades (Figure 6A). ML reconstructions estimate expression in all flowers at the base of the *L1O5, LHS1 *and *OSM5 *clades, with expression restricted to the upper flowers in *O. sativa OSM5 *and *OsLHS1, M. maximus LHS1, P. glaucum LHS1, S. bicolor LHS1 *and *Z. mays ZmM8 *and *ZmM14*. Expression restricted to the lower floret is only reported in maize *ZmM3*, and based on our reconstructions this partitioning of expression evolved relatively recently (since maize and sorghum last shared a common ancestor).

Together, these data do not support the subfunctionalization of *LHS1 *and *OSM5 *function into different regions within the grass spikelet following the *L1O5 *duplication event. However, our analyses are consistent with *LHS1 *functioning as a selector gene of the terminal flower in basipetally maturing spikelets of ehrhartoid and panicoid grasses.

## Discussion

*LHS1 *genes have been linked to the origin and diversification of the grass spikelet, but the role of its paralog, *OSM5*, has largely been overlooked. In this analysis we have used a combination of Bayesian phylogenetics, expression analyses, molecular evolutionary analyses, and MP and ML character reconstruction methods to investigate the evolutionary history of the *LHS1 *and *OSM5 *lineages of *SEP *genes. These analyses reconstruct a complex pattern of duplication and diversification and provide new insights into how these genes are expressed during spikelet and floret development in grasses.

### The *LHS1/OSM5 *duplication event maps to the base of the grass family

Our Bayesian phylogenetic analyses support the hypothesis that the *LHS1 *and *OSM5 *lineages are products of a gene duplication event at the base of the grass family. Although *LHS1 *co-orthologs were isolated from *S. angustifolia *and *P. latifolia*, the two earliest diverging members of the Poaceae, we were unable to isolate *OSM5 *co-orthologs from these taxa. The most parsimonious explanation hypothesizes independent losses of the *OsMS5 *co-orthologs in the two lineages. However, it is also possible that these *OSM5 *genes: 1) were expressed at a developmental stage not represented in our cDNA, 2) are too divergent to amplify using our degenerate primers, or 3) that the Bayesian tree has reconstructed the position of the *LHS1/OSM5 *duplication event incorrectly. We consider the independent loss hypothesis more likely based on three lines of evidence. First, the cDNA stocks used to isolate the genes using RT-PCR spanned diverse stages of inflorescence development, ranging from early branching through to floral organ initiation and maturation. Second, we were able to successfully amplify *LOFSEP *(Figure 1), *SEP3 *(Christensen and Malcomber, unpublished) and *AGL6-like *genes [49] from diverse other Poales using the same degenerate MADS primers and cDNA stocks. Third, the results of a Shimodaira-Hasegawa test [32] indicated that a constrained phylogeny where the *LHS1/OSM5 *duplication event occurred at the base of the BEP and PACCMAD clade and after the divergence of the Anomochlooideae and Pharioideae was significantly less likely (*P *< 0.001) than the unconstrained analysis with a duplication event at the base of the grass family.

A similar pattern of gene loss in *S. angustifolia *and *P. latifolia *was also reported in *FRUITFULL *(*FUL*) genes. In this analysis, Preston and Kellogg [50] were able to isolate *FUL2 *co-orthologs from *S. angustifolia *and *P. latifolia*, but not *FUL1*. Rice *OsFUL1 *and *OsLHS1 *both occur on chomosome 3, whereas *OsFUL2 *and *OsMADS5 *both occur on chromosome 6. Because *OSM5 *and *OsFUL1 *occur on different chromosomes we hypothesize that a more localized pattern of independent gene loss in the Anomochlooideae and Pharioideae produced the complex pattern of relationships within *FUL *and *LOFSEP *lineages rather than a pair of large chromosomal deletion events.

### OSM5 proteins are truncated relative to LHS1 and L1O5 proteins

Our molecular evolutionary analyses of the *LHS1, OSM5 *and *L1O5 *clade failed to find any evidence of positive selection (Additional file 4), but MP reconstructions estimated a cytosine to thymine substitution in the first codon position of the first amino acid immediately after the *ZMM3 *domain that converted a glutamine into stop codon and truncated OSM5 relative to the ancestral L1O5 proteins. This is a different hypothesis than proposed by Xu and Kong [19] who hypothesized that the insertion of a cytosine within the exon 8 of the rice *OsM5 *gene resulted in a frameshift and introduction of a premature stop codon. Our phylogenetic reconstructions of the *LOFSEP *data set estimate the insertion-deletion event within the rice *OSM5 ZMM3 *domain occurred relatively recently within the evolutionary history of the grass family (since ehrhartoid and pooid grasses last shared a common ancestor) and consequently did not coincide with the duplication event giving rise to the *OSM5 *lineage at the base of the grass family.

The C-terminal regions of MADS box genes are known to help regulate transcriptional activation, partner specificity, subcellular localization and/or the ability to attract interacting partners [8,41]. Deletion of amino acids 218 to 257 of the rice LHS1 protein, including both the *ZMM3 *and *OSMADS1 *domains, removed the ability of the protein to bind FUL1/OsMADS14 and FUL2/OsMADS15 [51] confirming a role for the C-terminus in regulating partner interactions. Cui *et al*. [18] reported that rice LHS1 and OSM5 proteins interact differently; OsLHS1 can homodimerize weakly and heterodimerize with the AGL11-like protein OsMADS13 and the SEP3 proteins OsMADS7 and OsMADS8, whereas rice OSM5 cannot homodimerize and cannot heterodimerize with OsLHS1, OsMADS7, OsMADS8 or OsMADS13. Whether rice LHS1 and OSM5 can heterodimerize with PAP2 has yet to be shown, but the discrepancy in binding abilities between OsLHS1 and OSM5 suggests that the truncation within the C-terminus of the OSM5 protein following the duplication event at the base of the grass family has had a profound effect on its ability to form multimeric complexes. The subtle phenotypic differences in rice *osmads5 *mutants with lodicules attached to the lemma and palea [17] suggests that OSM5 has a partially redundant role with other E-class genes during floral development. However, the inability of OSM5 to form complexes with the same set of proteins as OsLHS1 indicates that OSM5 is not (partially) redundant with OsLHS1. Together these data suggest that changes following the *LHS1/OSM5 *duplication have resulted in a new coding region function (or at least the loss of several functions currently performed by *LHS1*) for the *OSM5 *lineage and potentially some partitioning of function between *LHS1 *and *OsM5 *lineages.

### Complex patterns of expression of *OSM5 *and *LHS1 *genes

Our analysis of *OSM5 *and *LHS1 *mRNA expression profiles reveals a complex pattern of complimentary, overlapping and absent expression profiles within flowers (Figure 5) and in different regions of the inflorescence (Figure 6). Complimentary mRNA expression profiles of *OSM5 *and *LHS1 *occur in maize, where *ZmM3 *is restricted to the lower floret while *ZmM8 *and *ZmM14 *are only expressed in the upper floret, and within floral organs of certain sampled taxa, including: 1) the lemmas of rice and *P. glaucum *where *LHS1 *is expressed, but *OSM5 *is not, 2) the lodicules of *C. latifolium *where *LHS1 *is expressed, but *OSM5 *is not, 3) the stamens of rice and *P. glaucum *where *OSM5 *is expressed, but *LHS1 *is not, and 4) the carpels of *A. sativa, C. latifolium *and *S. bicolor *where *LHS1 *is expressed, but *OSM5 *is not, and in *P. glaucum *where *OSM5 *is expressed, but *LHS1 *is not. Several species also show overlapping expression profiles with *LHS1 *and *OSM5 *both being expressed in the lemmas and paleas of *A. sativa, S. bicolor *and *C. latifolium*, in the lemmas of *H. vulgare*, in the lodicules of *H. vulgare *and *P. glaucum*, and in the carpels of rice. Neither *LHS1 *nor *OSM5 *are expressed in the lodicules of *S. bicolor*, the stamens of *A. sativa *and *S. bicolor*, and the stamens and carpels of *H. vulgare *suggesting the E-class functional role in these structures is provided by either *PAP2 *or, more likely based on studies in rice [18], *SEP3 *co-orthologs. Taken together these data suggest a more labile pattern of expression evolution within the *LHS1 *and *OSM5 *clades than predicted by the classic model of subfunctionalization [1].

Expression profiles of *LHS1, OSM5, PAP2 *and the *SEP3 *co-orthologs *OSM7 *and *OSM8 *in rice *osmads7, osmads8, osmads7/8 *and *osmads1/5/7/8 *mutants provide additional insights into the plasticity of *SEP *gene expression in grasses and their roles during floral development. In *osmads7 *mutants, *LHS1 *(approximately 1.3 to 1.5× WT expression), *OSM5 *(approximately 2.5 to 3× WT expression), *OSM8 *(approximately 1.3 to 1.7× WT expression) and *PAP2 *(approximately 1.2 to 1.3× WT expression) were upregulated to compensate for the lack of *OSM7 *expression and in *osmads8 *mutants *LHS1 *(approximately 1.5 to 1.7× WT expression), *OSM5 *(approximately 1.8 to 2.1× WT expression), *OSM7 *(approximately 2.2 to 2.4× WT expression) and *PAP2 *(approximately 1.3 to 1.5× WT expression) were all upregulated and sufficient to produce mutant plants with only subtle phenotypes [18]. However, in *osmads7/8 *mutants even the upregulation of *LHS1 *(approximately 2.5 to 3.6× WT expression), *OSM5 *(approximately 1.6 to 2× WT expression), and *PAP2 *(approximately 1.7 to 1.8× WT expression) still produced severe mutant plants with two to four lodicules transformed into lemma and palea-like structures and three to seven longer and thinner sterile stamens [18], suggesting that the rice *LOFSEP *(*LHS1, OSM5 *and *PAP2*) genes are functionally divergent from the *SEP3 *co-orthologs and unable to complement the mutant. In *osmads1/5/7/8 *mutants, *PAP2 *expression ranged from 1.2 to 1.6× WT expression levels but even this increased expression was, again, unable to compensate for the lack of the other *SEP *genes with mutant plants having flowering delayed by three to four weeks, shorter inflorescences with increased branching, and florets with leaflike lemma, palea, lodicules, stamens and carpels [18]. Given the inability of LHS1, OSM5 and PAP2 to compensate for the lack of OSM7 and OSM8 in the mutant and the inability of PAP2 to compensate for the lack of SEP expression in the quadruple *osmads1/5/7/8 *mutant, these data point to either a direct or indirect negative feedback loop regulating expression among the different rice SEP genes, rather than a dosage effect among partially redundant genes [18].

This feedback loop could have compensated for the lack of *OsMADS5 *in rice *osmads5 *mutants which in turn resulted in plants with only subtle phenotypes. The different protein binding abilities of rice LHS1 and OSM5 suggests one (or more) of the other of the rice SEP genes is partially compensating for *OsMADS5 *in these mutants. Which of these other SEP genes, however, awaits further analysis of rice *osmads5 *mutants using real time RT-PCR.

## Conclusions

The grass *LOFSEP *genes *LHS1, OSM5 *and *PAP2 *all regulate aspects of grass inflorescence development, but the timing of the duplication events to produce the different lineages and the pattern of evolution within the different clades has not been fully investigated. In this analysis we used Bayesian phylogenetic methods to reconstruct relationships among 84 *LOFSEP SEPALLATA *genes that map the *LHS1 *and *OSM5 *duplication event at the very base of the grass family, whereas the *LHS1+OSM5 *and *PAP2 *duplication event maps to deeper within the monocot clade. MP reconstructions within the *LHS1 *and *OSM5 *lineage estimated a cytosine to thymine substitution converted a glutamine to stop codon immediately after the *ZMM3 *domain that truncated OSM5 relative to LHS1 and the L1O5 ancestor. Based on studies in rice, the truncation of the OSM5 protein and removal of the OSMADS1 domain has resulted in a different set of protein interaction partners [18,51]. This observation supports hypotheses of both subfunctionalization between LHS1 and OSM5 and neofunctionalization within the OSM5 lineage via a change in the coding region. ML character reconstruction analyses estimate a complex pattern of *OSM5 *and *LHS1 *expression evolution that is not consistent with a classic subfunctionalization model of partitioning following the gene duplication event. However, our analyses do support a hypothesis of recent *OSM5 *and *LHS1 *expression partitioning within the floral organs of *A. sativa, C. latifolium, H. vulgare, P. glaucum *and *S. bicolor*, and between the upper and lower florets of the two-flowered maize spikelet. How this complex pattern of gene expression evolution within the two lineages has affected morphological evolution in grasses has yet to be determined. The next step will be to expand expression analyses within the *OSM5 *lineage and couple these studies with investigations of *OSM5 *and *LHS1 *protein interactions and functional analyses of *osmads5 *and *lhs1 *single and double mutants in transformable grasses such as *Brachypodium distachyon *[52-54] and *Setaria viridis *[55].

## Abbreviations

*AsOSM5*: *Avena sativa ORYZA SATIVA MADS5*; BEP: Bambusoideae, Ehrhartoideae and Pooideae; BLAST: Basic Local Alignment Search Tool; *ClOSM5*: *Chasmanthium latifolium ORYZA SATIVA MADS5; FUL, FRUITFULL*; *HvOSM5*: *Hordeum vulgare ORYZA SATIVA MADS5*; *L1O5*: *LEAFY HULL STERILE1/Oryza sativa MADS5-like*; *LHS1*: *LEAFY HULL STERILE1*; LOFSEP: *Oryza sativa MADS1*/*LEAFY HULL STERILE1, Oryza sativa MADS5, Oryza sativa MADS34/PANICLE PHYTOMER2*, petunia *FLORAL BINDING PROTEIN9 *(*PhFBP9*) and *PhFBP23 *genes, and *Arabidopsis SEP1, SEP2 *and *SEP4; *ML: Maximum Likelihood; MP: Maximum Parsimony; NCBI: National Center for Biotechnology Information; *nsr*: *naked seed rice*; *OsLHS1*: *Oryza sativa LEAFY HULL STERILE1*; *OsMADS1*: *Oryza sativa MADS1*; *OsMADS34*: *Oryza sativa MADS34*; *OSM5*: *Oryza sativa MADS5*; *OsMADS7*: *Oryza sativa MADS7*; *OsMADS8*: *Oryza sativa MADS8*; *OsPAP2*: *Oryza sativa PANICLE PHYTOMER2*; PACCMAD: Panicoideae, Aristidoideae, Chloridoideae, Centothecoideae, Micrairoideae, Arundinoideae and Danthonioideae; PAML: Phylogenetic Analysis Using Maximum Likelihood; *PgOSM5*: *Pennisetum glaucum ORYZA SATIVA MADS5*; *PhFBP9*: *Petunia hybrida FLORAL BINDING PROTEIN9*; *PhFBP23*: *Petunia hybrida FLORAL BINDING PROTEIN23*; PP: Posterior Probability; RACE PCR: Randomly Amplified cDNA Ends Polymerase Chain Reaction; RT-PCR: Reverse Transcription Polymerase Chain Reaction; *SEP*: *SEPALLATA*; SH: Shimodaira-Hasegawa; *SbOSM5*: *Sorghum bicolor ORYZA SATIVA MADS5*; UTR: Untranslated Region; *ZMM3*: *Zea mays MADS3*...}

## Competing interests

The authors declare that they have no competing interests.

## Authors' contributions

AC isolated and aligned *LOFSEP *genes from non-model graminoid Poales, conducted *in situ *hybridization expression and PAML analyses, examined morphological development using SEM, and drafted the manuscript. SM conceived the study, conducted *in situ *hybridization expression, phylogenetic and character reconstruction analyses, and helped draft the manuscript. Both authors read and approved the final manuscript.

## Supplementary Material

Additional file 1 **Oligonucleotide primer combinations and primer sequences used to amplify LOFSEP genes from graminoid Poales**. Click here for file

Additional file 2 **Aligned LOFSEP Bayesian data set**. Click here for file

Additional file 3 **LOFSEP phylogenetic tree with posterior probabilities**. Click here for file

Additional file 4 **Summary of LOFSEP molecular evolutionary analyses using the CODEML package within PAML**. Click here for file
