# Supplementary material for: Duplication and diversification of the LEAFY HULL STERILE1 and Oryza sativa MADS5 SEPALLATA lineages in graminoid Poales
Source: EvoDevo. 2012 Feb 17;3:4. doi: 10.1186/2041-9139-3-4 (PMC3305426; doi:10.1186/2041-9139-3-4)
Supplement: Additional file 1 — Oligonucleotide primer combinations and primer sequences used to amplify LOFSEP genes from graminoid Poales. [file 2041-9139-3-4-S1.DOC]

Supplemental File 2. Semi-Nested PCR Strategy

Genus and species Target Gene Primers-Round 1 Primers-Round 2

| *Aristida purpurea* | *Al*PAP2 | MADS1F/POLYTADV | ZMM14 nested 2F/OSM34-R2 |
| --- | --- | --- | --- |
| *Avena sativa* | *As*OSM5 | MADS1F/POLYTADV | ZMM14 nested 2F/OSM5-R1 |
| *Avena sativa* | *As*PAP2 | MADS1F/POLYTADV | ZMM14 nested 2F/OSM34-R2 |
| *Chasmanthium latifolium* | *Cl*OSM5 | MADS1F/POLYTADV | ZMM14 nested 2F/OSM5-R1 |
| *Chasmanthium latifolium* | *Cl*PAP2 | MADS1F/POLYTADV | ZMM14 nested 2F/OSM34-R2 |
| *Cyperus involucratus* | *Ci*L1O5 | LHS1-32F/POLYTADV | RESTOSM34-324F/POLYTADV |
| *Danthonia sericea* | *Ds*PAP2 | MADS1F/POLYTADV | ZMM14 nested 2F/OSM34-R2 |
| *Ehrharta erecta* | *Ee*OSM5 | MADS1F/POLYTADV | ZMM14 nested 2F/OSM5-R1 |
| *Ehrharta erecta* | *Ee*PAP2 | MADS1F/POLYTADV | ZMM14 nested 2F/OSM34-R2 |
| *Elegia tectorum* | *Et*L1O5A | RESTSEP306F/POLYTADV |  |
| *Elegia tectorum* | *Et*L1O5B | LHS1-32F/POLYTADV | RESTOSM34-324F/POLYTADV |
| *Eleusine indica* | *Ec*OSM5 | MADS1F/POLYTADV | ZMM14 nested 2F/OSM5-R1 |
| *Eleusine indica* | *Ec*PAP2 |  |  |
| *Eriachne aristata* | *Ea*OSM5 | MADS1F/POLYTADV | SEP110F/POLYTADV |
| *Eriachne aristata* | *Ea*LHS1 | MADS1F/POLYTADV | SEP110F/POLYTADV |
| *Hordeum vulgare* | *Hv*OSM5 | MADS1F/POLYTADV | ZMM14 nested 2F/OSM5-R1 |
| *Joinvillea ascendens* | *Ja*PAP2 | LHS1-32F/POLYTADV | GRAMLHS1-125F/POLYTADV |
| *Leersia virginica* | *Lv*OSM5 |  |  |
| *Leersia virginica* | *Lv*PAP2 | MADS1F/POLYTADV | ZMM14 nested 2F/OSM34-R2 |
| *Lithachne humilis* | *Li*PAP2 | MADS1F/POLYTADV | ZMM14 nested 2F/OSM34-R2 |
| *Miscanthus sinensis* | *Mi*LHS1 | LHS1-32F/POLYTADV | SEP110F/POLYTADV |
| *Oryza barthii* | *Ob*LHS1 | MADS1F/LHS1-633R | LHS1-32F/LHS1-633R |
| *Oryza glaberrima* | *Og*LHS1 | MADS1F/LHS1-633R | LHS1-32F/LHS1-633R |
| *Oryza meridionalis* | *Om*LHS1 | MADS1F/LHS1-633R | LHS1-32F/LHS1-633R |
| *Panicum miliaceum* | *Pm*PAP2 | MADS1F/POLYTADV | ZMM14 nested 2F/OSM34-R2 |
| *Pennisetum glaucum* | *Pg*OSM5 | MADS1F/POLYTADV | ZMM14-124F/POLYTADV |
| *Pennisetum glaucum* | *Pg*PAP2 | MADS1F/POLYTADV | ZMM14 nested 2F/OSM34-R2 |
| *Pharus latifolius* | *Pl*LHS1 | LHS1-32F/POLYTADV | GRASSLHS1-386F/POLYTADV |
| *Pharus latifolius* | *Pl*PAP2 | LHS1-32F/POLYTADV | GRAMLHS1-125F/POLYTADV |
| *Setaria italica* | *Si*OSM5 | MADS1F/POLYTADV | ZMM14-124F/POLYTADV |
| *Setaria italica* | *Si*PAP2 | MADS1F/POLYTADV | ZMM14 nested 2F/OSM34-R2 |
| *Thamnochortus insignis* | *Ti*L1O5A | LHS1-32F/POLYTADV | LOFSEP142F/POLYTADV |
| *Thamnochortus insignis* | *Ti*L1O5B | LHS1-32F/POLYTADV | RESTOSM34-324F/POLYTADV |
|  |  |  |  |

Supplemental File 2. PCR Primer Sequences

Primer Name Primer Sequence (5’-3’)

| GRAMLHS1-125F | AGGTYGCBCTSRTCVTCTT |
| --- | --- |
| LHS1-32F | THGAGAACAAGATMARCMGGCA |
| LHS1-633R | TATCCAKCCRGATSGRMYRTGYTCATTSGGG |
| LOFSEP-142F | CTCTKCGAGTTCYCCAGC |
| MADS-1F | ATGGGTMGSGGSAAGGTGGAGCTGAAGCGG |
| OSMADS5-R1 | GTTRGCWTCAYBASSATGSCCACT |
| OSM34-R2 | CYGSAGRCGCTGGAAGAAGTG |
| POLYTADV | CCGGATCCTCTAGAGCGGCCGCTTTTTTTTTTTTTTTTT[V~Q] |
| RESTOSM34-324F | RCADAGRAATCTYCTWGGTGAGGA |
| RESTSEP-306F | GGAACAACWMCTGCAAGAAGC |
| SEP110F | TSCTSTGYGAYGCYGAGGT |
| ZMM14-124F | GAGGTCGCSCTCATCATC |
| ZMM14-nested 2F | GAGMTGARGCGGMTCGAGAACAAG |
|  |  |
