# Supplementary figures and images for: Duplication and diversification of the LEAFY HULL STERILE1 and Oryza sativa MADS5 SEPALLATA lineages in graminoid Poales
Source: EvoDevo. 2012 Feb 17;3:4. doi: 10.1186/2041-9139-3-4 (PMC3305426; doi:10.1186/2041-9139-3-4)

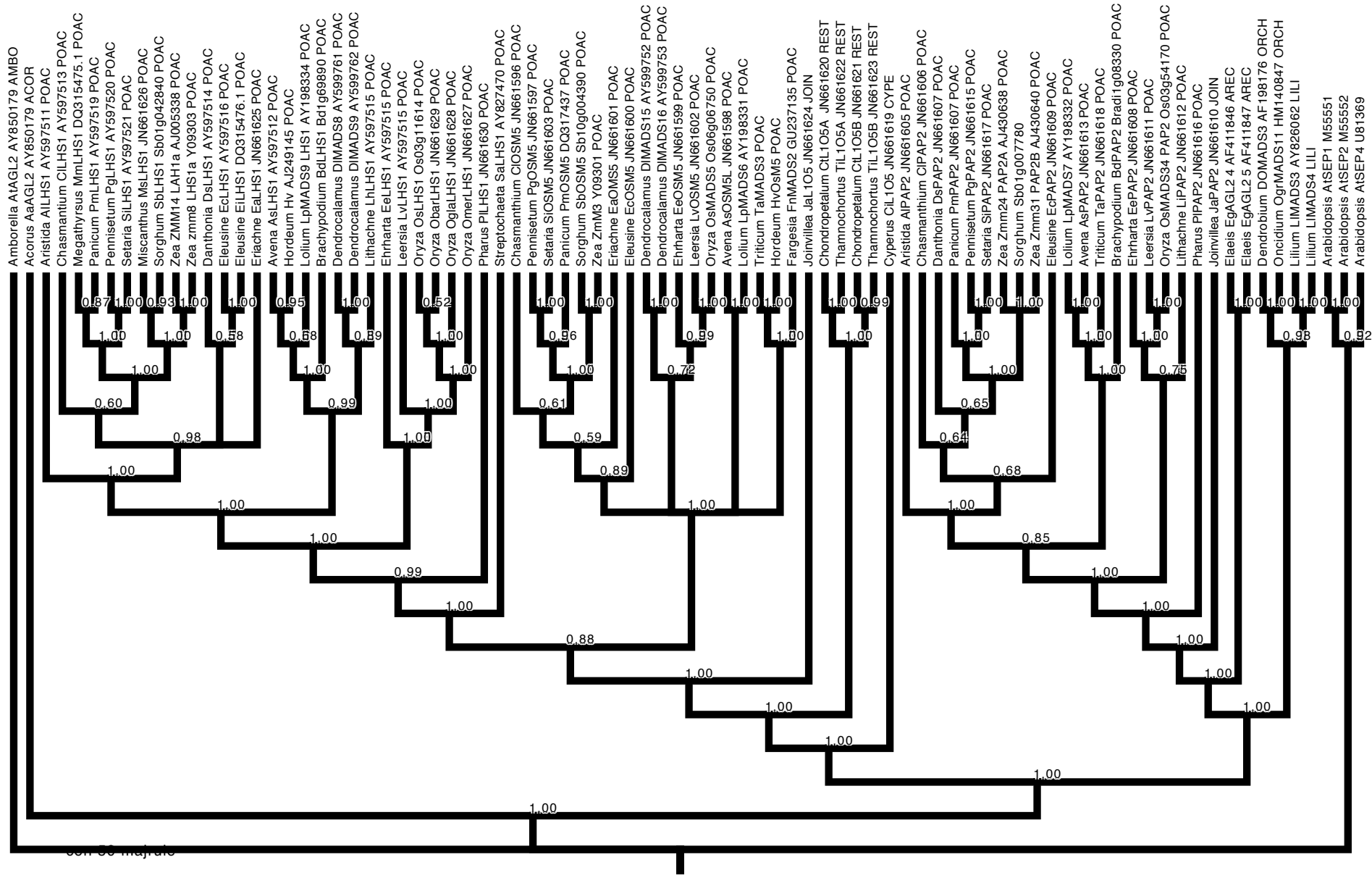

Supplement: Additional file 3 — LOFSEP phylogenetic tree with posterior probabilities. [file 2041-9139-3-4-S3.PDF]
