# Supplementary material for: Duplication and diversification of the LEAFY HULL STERILE1 and Oryza sativa MADS5 SEPALLATA lineages in graminoid Poales
Source: EvoDevo. 2012 Feb 17;3:4. doi: 10.1186/2041-9139-3-4 (PMC3305426; doi:10.1186/2041-9139-3-4)
Supplement: Additional file 4 — Summary of LOFSEP molecular evolutionary analyses using the CODEML package within PAML. [file 2041-9139-3-4-S4.DOC]

| Model | -ln | **ω** | 2**Δ**ln | Parameter estimates |
| --- | --- | --- | --- | --- |
| Model 0 (one ratio) | 3595.26 | 0.1656 |  |  |
| Model 1(nearly neutral) | 3562.43 | 0.305 |  | p_0_ = 0.817 p_1_= 0.192, **ω**_0_ = 0.139 |
| Model 2 (Kappa=3) | 3562.43 | 0.305 |  | p_0_ = 0.808, p_1_ = 0.165, p_2_ = 0.028, **ω**_0_ = 0.140, **ω**_1_ = 1.00, **ω**_2_ = 1.00 |
| Model 3 (discrete) | 3517.47 | 0.1814 |  | p_0_ = 0.46262 p_1_ = 0.335 p_2_ = 0.202 **ω**_0_ = 0.044 **ω**_1_ = 0.195 **ω**_2_ = 0.474 |
| Model 7 (beta) | 3520.64 | 0.1834 |  | p = 0.889, q = 3.881 |
| Model 8 | 3520.64 | 0.1835 |  | p_0_ = 0.9999 p = 0.88904 q = 3.881 (p_1_= 0.00001) **ω** = 3.14 |
| Model B null | 3525.76 | 0.1773 |  | p_0_ = 0.5833, p_1_ = 0.4167  **ω**_0_ = 0.0615, **ω**_1_ = 0.33943 |
| Branch 1 (LHS1+OSM5) -model A | 3562.43 |  | 0 | p_0_ = 0.80776, p_1_ = 0.19224, p_2_ = 0, p_3_ = 0, **ω**_0_ = 0.13961, **ω**_1_ = 1.00, **ω**_2_ = 1.00 |
| Branch 1 (LHS1+OSM5) -model B | 3525.28 |  | 0.966 | p_0_ = 0, p_1_ = 0, p_2_ = 0.583, p_3_ = 0.4167, **ω**_0_ = 0.061, **ω**_1_ = 0.3408, **ω**_2_ = 0, **ω**_3_ = 0 |
| Branch 2 (LHS1) - Model A | 3562.43 |  | 0 | p_0_ = 0.8078 p_1_ = 0.19244 p_2_ = 0.0 p_3_ = 0.0 **ω_0_** = 0.13961 **ω**_1_ = 1.00000 **ω**_2_ = 1.000 |
| Branch 2 (LHS1) - Model B | 3525.77 |  | 0.018 | p_0_ = 0.4611 p_1_ = 0.32933 p_2_ = 0.122 p_3_ = 0.0873 **ω**_0_ = 0.06145 **ω**_1_ = 0.33926 **ω**_2_ = 1.077 |
| Branch 3 (OSM5) - Model A | 3562.43 |  | 0 | p_0_ = 0.80776 p_1_ = 0.19224 p_2_ = 0.0 p_3_ = 0.000 **ω**_0_ = 0.13961 **ω**_1_ = 1.00000 **ω**_2_ = 1.0000 |
| Branch 3 (OSM5) - Model B | 3524.51 |  | 2.500 | p_0_ = 0.00 p_1_ = 0.000 p_2_ = 0.583 p_3_ = 0.417 **ω**_0_ = 0.06173 **ω**_1_ = 0.000 **ω**_2_ = 0.000 |

No sites identified using Bayesian Empirical Bayes (BEB) criteria as potentially under positive selection
